# Guidelines and mindlines: why do clinical staff over-diagnose malaria in Tanzania? A qualitative study

**DOI:** 10.1186/1475-2875-7-53

**Published:** 2008-04-02

**Authors:** Clare IR Chandler, Caroline Jones, Gloria Boniface, Kaseem Juma, Hugh Reyburn, Christopher JM Whitty

**Affiliations:** 1 London School of Hygiene and Tropical Medicine, Keppel Street, WC1E 7HT, London, UK; 2 Joint Malaria Programme, Kilimanjaro Christian Medical Centre, P O Box 2228, Moshi, Tanzania

## Abstract

**Background:**

Malaria over-diagnosis in Africa is widespread and costly both financially and in terms of morbidity and mortality from missed diagnoses. An understanding of the reasons behind malaria over-diagnosis is urgently needed to inform strategies for better targeting of antimalarials.

**Methods:**

In an ethnographic study of clinical practice in two hospitals in Tanzania, 2,082 patient consultations with 34 clinicians were observed over a period of three months at each hospital. All clinicians were also interviewed individually as well as being observed during routine working activities with colleagues. Interviews with five tutors and 10 clinical officer students at a nearby clinical officer training college were subsequently conducted.

**Results:**

Four, primarily social, spheres of influence on malaria over-diagnosis were identified. Firstly, the influence of initial training within a context where the importance of malaria is strongly promoted. Secondly, the influence of peers, conforming to perceived expectations from colleagues. Thirdly, pressure to conform with perceived patient preferences. Lastly, quality of diagnostic support, involving resource management, motivation and supervision. Rather than following national guidelines for the diagnosis of febrile illness, clinician behaviour appeared to follow 'mindlines': shared rationales constructed from these different spheres of influence. Three mindlines were identified in this setting: malaria is easier to diagnose than alternative diseases; malaria is a more acceptable diagnosis; and missing malaria is indefensible. These mindlines were apparent during the training stages as well as throughout clinical careers.

**Conclusion:**

Clinicians were found to follow mindlines as well as or rather than guidelines, which incorporated multiple social influences operating in the immediate and the wider context of decision making. Interventions to move mindlines closer to guidelines need to take the variety of social influences into account.

## Background

Whilst malaria remains the most important diagnosis in children in most of Africa, and in peripheral settings is often missed, the overdiagnosis of malaria at hospitals and health centres has been widely reported [1-3]. Overdiagnosis involves both the prescription of antimalarials to patients without evidence of malaria parasitaemia and the frequent absence of treatment for alternative causes of disease [4]. Many of the alternative diagnoses in children are likely to be bacterial and potentially fatal [5]. The practice also exposes patients to unnecessary side effects and treatment costs [6] and raises concerns for the risk of resistance to and sustainability of subsidy for new and more expensive antimalarials [7]. It makes assessment of the impact of new and effective malaria interventions very difficult as the reported burden of disease from formal healthcare remains the same even if the control interventions mean true disease incidence is reducing.

While clinical algorithms are sensitive they have low specificity for malaria diagnoses [8] and most national guidelines, including those in Tanzania, state that where malaria testing is available alternative causes of disease should be considered in the light of negative results [9]. However, the over-diagnosis of malaria continues in spite of evidence that restricting antimalarials to true test-positives has been shown to be safe in areas of low endemicity [10], where the majority of antimalarials are currently used, and in spite of the deployment of new rapid diagnostic tests (RDTs) [11,12]. In Tanzania, malaria was found to be over-diagnosed with equal frequency whether the clinician used an RDT or conventional microscopy [11]. An understanding of the reasons underlying current behaviour is urgently needed in order to inform strategies to improve the prescription of antimalarials.

Quantitative studies exploring malaria treatment patterns suggest that clinical symptoms are not the only determinants of practice. Factors such as consultation length [13], time of day [14], number of clinicians working, patient load, clinician sex and individual facility affect decision making. These studies also suggest that social factors, that are not easily measured quantitatively, may have had an affect on the treatment decisions that were explored.

Sociologists have long recognized that the social context of clinical practice affects prescribing [15,16] and broader influences on clinical care are now recognized by policy makers [17]. Rowe *et al *[18] reviewed potential determinants of inadequate health worker performance in low resource settings, and identified, alongside patient factors, influences from the environment and administration of the health facility as well as the community and the wider economic and political spheres. However, relatively few studies have explored the social environment of specific clinical decisions in developing countries, particularly in the hospital setting [19] and the reasons for malaria over-diagnosis have not been systematically explored.

Ethnographic methods have proven useful in describing the cultures of healthcare provision, and a limited number of such studies have been undertaken in developing country hospitals [20,21]. The extensive observation of events and naturally occurring discussions enables the exploration of unreported elements relevant to the decision-making process of respondents [22]. As such, ethnographic studies of the over-prescription of antibiotics in developing countries have identified complex interactions of different spheres of influence [23,24]. Such findings and frameworks are useful in informing behaviour change interventions [25]. An understanding of the influences on malaria diagnostic and treatment decisions is essential if antimalarials are to be targeted to those who most need them and alternative diagnoses treated. This study set out to provide information on this.

## Methods

### Setting

Tanzania has a stable government run health service throughout its 20 regions. Each region is divided into districts, with a government-run or mission-government co-run district hospital as well as other government and private health facilities. This study was conducted in the Kilimanjaro and Tanga regions of Tanzania. Overall, malaria transmission in Tanzania is high, although this varies across regions. Malaria transmission in the Kilimanjaro region is lower than in the Tanga region [26]. The study took place in two hospitals, one in each region, with malaria the single most common diagnosis at both hospitals. At the time of study, 31% outpatients and 18% admissions were diagnosed with malaria at the hospital in the Kilimanjaro region (HI); 37% outpatients and 53% admissions were diagnosed with malaria at the hospital in Tanga region (HII). The prevalence of HIV also varies across regions and districts but overall estimates from surveillance data at antenatal clinics was 8.7% and amongst blood donors was 7.7% in 2004 [27]. National treatment guidelines are based on key diseases (such as malaria and HIV/AIDS), and do not cover all febrile illness although the Ministry of Health's recent adoption of the WHO promoted 'Hospital Care for Children' [28] extends clinical guidelines to a syndromic approach which covers the treatment of all common paediatric febrile illness in settings with limited resources. Both of these guidelines state that patients with fever should be tested for malaria where possible and that in the case of negative results, alternative diagnoses and treatment should be considered. Where other diagnoses are ruled out, malaria treatment should be prescribed. The national malaria treatment guidelines do not detail alternative diagnoses whereas these are comprehensively described in the WHO hospital paediatric care guidelines. The study took place over a 12 month period between 2006 and 2007.

### Ethnography

The main method of study was ethnography in two district hospitals with three months fieldwork undertaken at each location. Over-diagnosis of malaria had previously been documented at both hospitals, which had similarly high patient loads but represented contrasting settings: hospital 1 (HI) was co-funded and co-run by the Catholic Church and the government and was in an area of low transmission for malaria; hospital 2 (HII) was government funded and run and was in an area of high transmission. The fieldwork was conducted by CC with the support of a local non-medical research assistant able to translate between English and KiSwahili at each hospital. The ethnography involved non-participant observation of clinician activities during their shifts, clinician-patient interactions during outpatient and inpatient consultations and interactions with colleagues during the working day and in daily clinical meetings. Notes of these observations were made using a series of notebooks and a piloted data collection form for consultations. With the consent of clinicians and patients, around 10% consultations were also tape recorded. The ethnographer (CC) was conversational in KiSwahili but research assistants, often present during consultations and meetings, helped with nuances of the language and with translating tape recordings of consultations. The ethnography also involved informal interviews using open ended questions, often built from issues raised by conversations or observations of daily practice or specific cases. In-depth interviews focussing on perceptions of malaria, malarial testing and treatment as well as motivation factors were held with all clinicians observed during patient consultations as well as with administrative, laboratory and nursing staff. These interviews were conducted in English and were tape recorded if circumstances permitted, with the consent of the participant. Clinicians were selected for observation if they worked on the paediatric ward, in the paediatric outpatient or general outpatient departments, and frequency of observation of each clinician was in proportion to the frequency of consultations conducted by each clinician according to hospital records. Clinicians included clinical officers (COs) with three years training; assistant medical officers (AMOs) with a further two to three years training; or medical officers (MOs) with full medical training. Informed written consent was obtained from all staff participating in the study, and on obtaining consent the clinicians were also invited to fill an enrolment questionnaire of demographic and work history details. Informed verbal consent was requested from all patients or caretakers at the start of each consultation observed.

A key methodological issue was the role of the ethnographer (CC) in the hospital. Observation, recording of observations and conversations and conducting longer interviews did not fit with either clinical or patient role definitions. However, an advantage of being an outsider to both the medical profession and the local culture was a justification for the numerous enquiries about everyday phenomena and practices. Practice is known to change in the presence of observers, the Hawthorne effect, but this is also known to reduce rapidly over time [29] so that for most of the three month observation period this effect may have been reduced. The process of informing and obtaining consent from participants throughout the study may have increased awareness of being observed, but any impact on behaviour is likely to have been towards perceived best practice [30].

### Training college interviews

Subsequent to preliminary analysis of the ethnographic study, semi-structured in-depth interviews were held with students and tutors at a clinical officer training college (COTC) in northeast Tanzania over a two week period. The COTC was situated in an area of low malaria transmission, but intended to prepare students for a range of epidemiological settings. These interviews sought to establish whether themes generated in the ethnography stage were consistent with current teaching or student/tutor beliefs. Topics included perceptions of malaria, the use of laboratory investigations and the use of antimalarials along with an exploration of teaching methods and processes. All tutors present at the COTC at the time of the study were invited to participate and appointments arranged for interviews in advance. Students were recruited using convenience sampling, by selection from classrooms during periods of private study. Equal numbers of students from each of the first, second and third years were sought for interview and recruitment ended when no new responses to the topics of interest emerged. All interviews were tape recorded. Participants gave written informed consent to participate in the study.

### Ethics

Ethical approval was granted for the study from the National Institute of Medical Research in Tanzania (XIII/427) and from the London School of Hygiene & Tropical Medicine (4048).

### Analysis

Analysis of the ethnographic data was undertaken using Nvivo7 software (QSR International) and was ongoing during the data collection period. Transcripts and field notes were coded into broad categories daily, supplemented by frequent more detailed coding sessions, allowing the data to guide coding rather than imposing a coding scheme [31]. Concepts were developed from the codes, building a theoretical framework describing influences on behaviour that included actions, interactions, reported behaviour and spoken or unspoken norms or standards. Analysis of the interview data from the COTC followed the same methodology as for the ethnographic analysis.

## Results

### Study participants

During the ethnographic fieldwork, 2,082 consultations and 80 clinical meetings were observed. A total of 34 in-depth interviews with clinicians and 14 interviews with administrative, laboratory or ward staff were held. Table 1 describes the sample of clinicians observed and interviewed as well as the patients observed during consultations. There were more clinicians working at HI where clinicians were also older and more likely to originate from the area around the hospital. Fewer consultations were observed at HI where the patient load was lower and where, as a consequence, more adult consultations were observed. Outpatient consultations were the focus of the study as this is the point where the majority of diagnostic decisions are made for both admitted and non-admitted patients. A smaller percentage (27% HI, 16% HII) of consultations were observed during ward rounds, with a focus on paediatric wards which have the highest rates of malaria admissions. In-depth interviews formed the bulk of the interview material, but field notes detailing informal conversations, interactions and clinical meetings were also extensive. Six tutors at the COTC were invited for interview: five accepted and one declined due to workload pressures. 10 students were interviewed. Table 2 describes the COTC study sample.

**Table 1:** Clinicians and patients participating in ethnography

	**HI**	**HII**
Type of hospital	Mission designated	Government
Malaria transmission intensity	Low	High
**Clinicians**	% of **21 **participating clinicians	% of **13 **participating clinicians
Age group		
40 + years	66.7	30.8
Sex		
Male	66.7	76.9
Medical qualification		
MO	4.8	0
AMO	19.0	7.7
CO	76.2	92.3
Year of graduation (most recent qualification)		
Since 2000	38.1	61.5
Number of years worked at hospital		
<2 years	33.3	53.8
3–9 years	22.2	30.8
10+ years	44.5	15.4
Employer		
Government/District	71.4	100.0
Mission	28.6	0.0
Number of in-service training seminars attended in last 12 months		
1 or none	25.0	38.5
2 or more	75.0	61.5
Originate from area around hospital	50.0	15.4

**Patients**	% of **673 **patients	% of **1,409 **patients
Age under 5	47.4	81.0
Outpatient consultations	73.0	83.9

**Table 2:** Participants of COTC interviews

	**N**	**n female**	**Age range**
Tutor: Medical Doctor	1	0	51
Tutors: CO/AMO	3	0	41 – 65
Tutor: Nurse	1	1	35
Students: First year	3	1	19 – 28
Students: Second year	3	1	22 – 35
Students: Third year	4	1	23 – 30

### Observed practice

Malaria diagnoses were frequent at both hospitals and antimalarials were often observed to be prescribed to patients without testing blood for malaria parasites, with negative test results and even patients without fever (Table 3). Antimalarials were prescribed more frequently at HII (to 47% of patients) than HI (to 19% patients). This may follow the epidemiological distribution of malaria, with HII in an area of higher transmission than HI, also reflected in the slightly smaller proportion of patients with laboratory confirmed malaria at this hospital (5.3% compared to 10.9% at HI). However, the high proportion of antimalarial prescriptions to afebrile and laboratory unconfirmed cases follows neither clinical rationale, guidelines nor, particularly at HI, epidemiological evidence.

**Table 3:** Pattern of antimalarial prescription at ethnography hospitals

	HI	HII
Percentage patients prescribed antimalarials	19.0 (of 673)	46.7 (of 1409)
Percentage febrile of those prescribed antimalarials	38.3 (of 128)	72.0 (of 658)
Percentage patients tested for malaria	30.0 (of 673)	25.8 (of 1409)
Percentage malaria test positive of those prescribed antimalarials (vs negative or untested)	10.9 (of 128)	5.3 (of 658)

The prior presumption of malaria amongst both children and adults appeared high. Malaria diagnosis was routine with patients presenting with symptoms including any of fever, vomiting, headache or joint pain either directly given an antimalarial prescription or a test and an antimalarial prescription (often not following the test result). Often several patients seen in series would be prescribed antimalarials in very short consultations involving few if any examinations or other laboratory tests. In contrast, consultations ending in non-malarial diagnoses took statistically significantly longer than when malarial diagnoses were made (median 5 minutes compared to median 4 minutes for malaria diagnoses, Mann-Whitney test p = 0.017). Guidelines and processes for the easy diagnosis and treatment of diseases alternative to malaria were poor or missing at the hospital and national levels.

### Spheres of influence

Analysis identified a number of factors affecting malaria over-diagnosis which could be grouped into four spheres of influence. The first is the influence on clinicians of training in the context where malaria is strongly promoted. The second influence is pressure from peers and the third perceived patient preferences. The forth influence on clinicians is physical and organisational diagnostic support at the local level, including the availability, management and supervision of equipment and of staff. Each of these spheres is discussed below, and Table 4 summarizes how these spheres of influence weight both towards making malaria diagnoses and away from making alternative diagnoses.

**Table 4:** Influences and mindlines for malaria over-diagnosis

**Sphere of influence**	**Motives for malaria diagnosis**	**Motives for not treating alternative diagnoses**	**Motives for treating alternative diagnoses**	***Mindlines***
**Patient outcome**	Patient likely to be cured if malaria parasites present		Patient likely to be cured if no malaria parasites present at low endemicity, or even if parasitaemic at high endemicity (co-morbidity)	

**Diagnostic support**	Easy and quick to diagnose and treat	More complicated diagnosis and treatment		***Malaria is easier to diagnose than alternatives***
	Perceived as easily recognisable	Alternative diseases perceived as less specific		
	Fewer tests needed for confirmation	Increased number and complexity of tests (e.g. lumbar puncture)		
	Feel confident to diagnose clinically	Clinical diagnosis less clear, may need to wait for test results		
	Laboratory test results may be incorrect (due to resource problems, staff skills) or the parasites may be hidden			
	Well established process of malaria diagnosis and treatment	No set process: if time short or motivation low may be easier to take established path		
	No retribution for over-diagnosing malaria	Lack of supervision or regular advice to consider differential diagnoses		
**Disease promotion and training**	Well known disease with frequent training available	Less well known diseases, less training available		
	Guidelines are malaria specific	Few guidelines for alternative diseases		
**Patient preferences**	Perceived as preferable to patients	Fear of patient complaints if don't test or treat for malaria		
	Perceived as acceptable to patients: high profile, low-stigma disease	More explanation necessary for patients who may prefer the familiarity of malaria		***Malaria is a more acceptable diagnosis***
**Peer pressure**	Perceived as acceptable to peers who also see over-diagnosis as preferable to missing malaria	Alternative diagnoses may require clinicians to justify themselves		
**Disease promotion and training**	Malaria promoted by public health campaigns as most important disease	Alternative diseases less often promoted resulting in lower profile		***Missing malaria is indefensible***
	Training emphasises malaria over alternative diseases	Alternative diseases taught in theory more than practice		
	Indefensible to miss malaria, perceived as most important disease	More defensible to miss alternative causes of disease		

### Training and promotion of the importance of malaria

Public health messages promote malaria as the most common and important disease to diagnose and treat in Tanzania. Contrary to epidemiological evidence, clinicians reported that malaria was increasing in prevalence in Tanzania and that it was the most important diagnosis at their hospitals even at low transmission. The influence of these messages, together with interpretation of guidelines, built within the clinicians an understanding that malaria should always be treated as the first priority.

410(Clinical Officer, CO, Hospital II, HII) "*These guidelines say that a negative slide cannot rule out malaria*"

408(CO, HII) "*It is according to the government that if the slide is negative but the patient has the signs and symptoms of malaria then you have to treat that patient with antimalarials*"

Clinicians frequently spoke of their fear of missing a malaria diagnosis, describing this as a potentially fatal mistake,

207(CO, HI) "*If you will say that I should not treat this patient until the blood slide is positive you will kill the patient*"

204(CO, HI) "*We know in this area almost most of the people [researchers] they say that there is no malaria, but if you don't treat our people with antimalarials we will lose a lot of people, especially the children*..."

This fear remained in the face of low probability of malaria, in areas of low endemicity and/or when a malaria test was taken and the result was negative,

410(CO, HII) "*She came with fever and I sent her for a malaria slide but it was negative. But she is still having fever so I prescribed antimalarials. Suppose you say it is not malaria and send her home, what do you say in three days when she comes back and it is worse? Especially in our setting that is endemic for malaria*" (discussing a febrile paediatric patient. Amodiaquine was prescribed with paracetamol).

Interviews with tutors and students at a clinical officer training college suggested these fears were consistent with current teaching, which emphasized malaria above all other disease diagnoses.

601(Tutor, COTC) "*Even if it is negative, the patient has got a high temperature has got all the clinical sign of malaria you can't say you are not going to treat that patient and you say you are going to give paracetamol of course you might see that he is doing well but still he is sick, I don't think it is good to wait until it get complicated like some malaria or renal failure or somebody with pregnancy you don't have to wait until she get an abortion then you say let me give Fansidar [SP]*"

### Peer pressure

Malaria over-diagnosis was common across different clinicians and hospitals suggesting a pattern of behaviour that was constantly being re-inforced by the actions and reactions of other clinicians. In discussing malaria diagnostic decisions, clinicians frequently relied on a collective rather than their individual response. For example, the following respondent used the term 'we' in recourse to medical (but epidemiologically rare) justifications for malaria test-negative antimalarial treatment,

CC: "*Tell me about slide negative malaria: how is it possible?*"

106(CO, HI): "*It is very possible for a person with malaria to have a slide negative*"

CC: *How*?

106: "*It is pathophysiology: malaria rotates around the liver so when you take the blood slide it can often not find it*"

CC: "*How many slides are negative here?*"

106: "*It is about almost 50–50 to get a slide negative as slide positive malaria*"

CC: "*Do you treat slide positive differently to slide negative malaria?*"

106: "*No we do not give them different management. The most important thing is symptoms not investigations. If they have got symptoms, we treat regardless of whether it is positive or negative*."

Furthermore, the narrative of several clinicians at HI regarding findings of low malaria transmission in the area by researchers in the previous few years suggested that there was a mutual agreement, possibly tacit, to dismiss the results:

113(CO, HI): "*... research was done and the conclusion was rather peculiar saying 'what you are treating isn't malaria you are treating febrile conditions'. We were very astonished because all these cases we are giving malaria treatment and they get cured. They said 'no but this is febrile condition'. We were very much astonished to hear that but we didn't stop giving antimalarials ... we insisted that this is malaria regardless of the slide being negative, but it is malaria because our patients have been all treated with antimalarials and nothing else and they get cured*."

Peers were not observed to raise criticism over the high frequency of malarial diagnoses. For example, observation of daily clinical meetings when cases were reviewed suggest that it was expected that antimalarials would be prescribed as first course of action for most patients with a medical complaint regardless of negative malaria test results or no history of fever. However, these meetings also demonstrated that decisions were not made within a blame-free culture. Criticism of decisions, especially those made by clinical officers, was frequent,

CC: "*How do you feel about the clinical meeting? Is it useful?*"

Clinician 304(CO, HII): "*Ah! You know there are really two groups – those AMOs and then us COs and they like to just criticize us. That is what happened this morning. It isn't useful... They are just showing that we have done this or that wrong. That is why many of us clinical officers just don't go to the meeting. They don't like it*."

The fear of humiliation in front of colleagues appeared to be a driving factor in clinicians' motives for particular decisions, and it was clear that missing apparently obvious diagnoses, especially malaria, would invoke such public criticism. Corresponding retribution for missing alternative febrile diagnoses was not apparent.

### Perceived patient preferences

Clinicians reported feeling under pressure from patients,

112(CO, HI) "*these patients they are telling you what they want, you are not telling them!"*

107(CO, HI): *" ... sometimes they come saying I have this or this or I want this and you have to say, "what are your symptoms, are you sick?". but sometimes they are forcing you to do what they want"*

304(CO, HII): *"They are demanding. Some come with a diagnosis like "I have got malaria and I want a bs [malaria blood slide]"*

A quarter of clinicians said they were afraid patients might complain if they did not give them antimalarials. However, patients were rarely observed to directly ask for antimalarials, though sometimes they did request malaria tests.

### Diagnostic support

At interview, hospital clinicians described malaria as easy to diagnose clinically. They were more confident in their clinical judgement than in laboratory evidence and stressed that antimalarials should not be restricted to test-positive patients. The same views were given by tutors and also students at the COTC.

602(Tutor, COTC) *"60% of the diagnosis is done by proper history taking and examination, this is what I teach them. And it is true that you can have 60% of the diagnosis when you take properly the history and do appropriate examination then 40% is done by proving by laboratory means... sometimes they can have a negative blood slide but if all the symptoms maybe 60% make you think of malaria then we say we treat clinically."*

However, while clinicians were confident in their diagnostic abilities, they also received little support for alternative diagnoses. Diagnostic support can be seen as both physical and organisational and the diagnostic support for clinicians at the two hospitals in this study was constrained both physically and in organisational terms such that malaria became the easiest diagnosis to make. For example, one obvious alternative cause of severe symptoms (high fever, convulsions, reduced level of consciousness) is meningitis, but clinicians were observed to be reluctant to carry out lumbar punctures, a more complicated and time consuming test, which was performed less than 10 times during the observation period. In addition, HIV related disease might be considered but would require far greater effort on the part of the clinician, with the need for counselling contributing to diagnostic barriers,

403(AMO, HII): *"There are other things that are now arising, with this new HIV/AIDS*... *clinicians don't think symptoms are AIDS related: they just think malaria, pneumonia and diarrhoeal disease, rather than the HIV. But if they were to think, there would be many diagnoses... You know according to our rules, they can't screen without counselling, so they have no authority to test without patient consent. So even if they think "I should think about HIV for this patient" they cannot. If we could screen like we do with malaria it could be easier, but the policy is not yet. It is the stigma of the disease still... If you see someone who you are doubting of the disease you could just send for the test. So they simply file that we are dealing with malaria or pneumonia."*

Trust in laboratory test results was raised by some clinicians. There was a consensus amongst interviewees that the quality of laboratory test results was dependant upon resource factors such as availability of electricity and working equipment, together with the skill of laboratory technicians or attendants.

406(CO, HII): "*...the lab is not well equipped, not only the equipment but the staff*"

CC: "*Would it be better to have more staff in the lab and then you could test more patients?*"

406:" *Actually, it is not more staff, but more trained staff. It might just be an attendant who is looking at the slides and then they just write "positive" when it is negative."*

Organizational factors also affected reliance on tests. Delays in receiving test results were common: antimalarials were prescribed to 56% patients who were tested for malaria before the results were received.

204(AMO, HI): "*When I'm doing ward round I find the child is there and maybe for three days and the investigations were ordered and I ask the mother did you pass the laboratory and [she] says yes I did. The child was taken blood so I have to chase the one who I'm with saying I'm not going on until you give me the form*."

General management of resources and staff was observed and reported to be poor. Equipment shortages affected decision-making, for example, the low number of thermometers available at the time of consultations (in spite of a good supply to the store) led to infrequent measurement of temperature. The lack of concern shown by management about issues such as these had a knock-on effect on willingness to pursue best practice and made easy diagnoses a more attractive option. Similarly, poor management of staff in terms of workloads affected practice:

402(AMO, HII): "*The patient load is true too: if so many patients are there we can be very harsh and don't do a full history*"

Supervision of diagnostic practice was limited and there was little support for improving diagnoses.

CC:*"Is there ever any supervision of actual practice of diagnostic and treatment decisions?*"

Clinician 404(CO, HII):" *I have never observed something like that... when someone mismanages a patient they don't even call that clinician to say this is how we treat this or that. For example [another clinician] last week admitted a child and the doctor in charge called her in but instead of telling her what was wrong with the management she just criticized her. You know, we don't have treatment guidelines, we are just working to our own knowledge. Even we don't know what drugs are here. Sometimes I may prescribe drugs that are not available or are expensive and the patient has to go far to get the drugs when maybe it was an emergency. It would be good to have a list that said "this is out of stock, better use this for now". You know, the management of this hospital is like it is absent. What I experienced before a number of deaths here result from our own misconduct."*

Even if clinicians were motivated to think of alternatives, dissemination of teaching or materials regarding up-to-date guidelines and processes relied on the staff returning from workshops to carry out teaching sessions. This practice was rarely observed. Staff complained of frustration, both at the lack of dissemination of information and the selection of staff for training opportunities. Selection was perceived as biased and thus provision of training was observed to be a demotivator to staff who had not been selected.

Clinician 207(CO, HI): *"You know, they only select the same persons to go to seminars."*

CC: "*Oh, really*?"

Clinician 207: "*Yah he will be the only one, he will be the one who will go the seminar of this, the next seminar he will go the same person, next seminar will go the same person, hah! in reality they're supposed to train everybody when they come back...but it is not done*."

Clinicians admitted that part of the reason for wanting to attend workshops was for extra payments received and this appeared to outweigh the goal of learning in some cases, demonstrated by complaints or refusals to attend when small or no monetary compensation was given.

Of the few posters and guidelines displayed at the hospitals, the majority were malaria related and most research previously conducted at the hospitals had been malaria related.

Diagnostic support at the hospital level, with established malaria diagnostic practices reinforced by poor resource and staff management and poor supervision and guideline accessibility, together with an emphasis in the working environment on malarial diagnoses, weighted decisions towards malarial rather than non-malarial diagnoses in the hospitals observed.

### Mindlines

**Figure 1:**
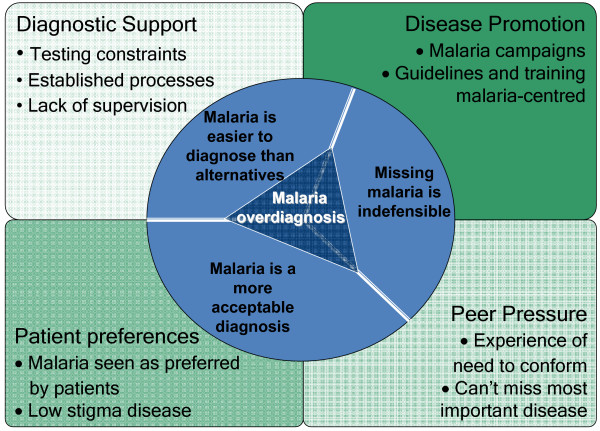
Mindline model for the over-diagnosis of malaria.

Guidelines available to clinicians working at district hospitals state that all febrile patients should be tested for malaria when testing is available and that those with negative results should be considered for alternative causes of disease and only treated with antimalarials if these alternative causes are ruled out. Actual practice varied from guidelines with patients frequently treated for malaria without testing or with negative test results. A number of non-clinical factors were found to influence this preference for malaria diagnosis and treatment which were systematic, but differed from the guidelines. Analysis of these influences suggests they contribute to the construction of rationales that inform clinical behaviour and which clinicians conform to in contrast to clinical guidelines. Gabbay and le May [32] described such rationales as 'mindlines' for prescribing in general practice in England, defined as "collectively reinforced, internalized tacit guidelines, which were informed by brief reading, but mainly by their interactions with each other and with opinion leaders, patients ... and from other sources of largely tacit knowledge that built on their early training and on their own and their colleagues' experience"(p.1015). Three such mindlines could be described to lead to malaria over-diagnosis in this study: that malaria is easier to diagnose than alternative diseases; that malaria is a more acceptable diagnosis; and that missing malaria is indefensible in this setting (Figure 1 and Table 4). These mindlines appear to have evolved through a combination of the relative difficulty of diagnosing and treating alternative causes of diseases that present with similar symptoms to malaria, the lack of supervision to ensure these diseases were not missed and the teaching of clinicians that their clinical judgement was the best tool for malaria diagnosis. Thus malaria was seen as the easiest and safest diagnosis to make in this setting where constraints on equipment and time are common. Additionally, malaria was an acceptable diagnosis both to patients, who were seen to prefer malarial diagnoses especially in contrast to other potential candidate diseases such as HIV, and to peers who accepted malarial diagnoses readily and reinforced the paradigm that malaria is easily recognizable and should not be missed. The fear of missing malaria under lay many of the clinicians' responses regarding antimalarial prescribing. The emphasis on malaria from both public health messages and clinical training appears to result in, or reinforce, the idea that to miss malaria is indefensible, and more so than missing other potentially serious diseases. The mindlines described appear to be shared by clinicians as a group although individuals could adhere more or less strongly to them depending upon their experiences with, and responses to, the different spheres of influence described.

## Discussion

Malaria over-diagnosis is common even when malaria testing is available in district hospital settings and interventions to improve clinical decision making are urgently needed to target new and more expensive antimalarials to those who most need them. This study provides evidence that the over-diagnosis of malaria in two district hospitals in north-eastern Tanzania derives from poor diagnostic support (resource management and lack of supervision), peer group pressure, perceptions of patient preferences and from public health and training promotion of malaria as the most important disease.

Poor resource management and a lack of supervision have been reported elsewhere in Africa [1,33] and poor supervision has been linked to resumption of prior behaviour after initial improvement due to training [34]. That these factors, together with a high workload, too few staff and infrequent or unequal opportunities for training, contributed to the demotivation of staff in our study is consistent with findings from Africa [35] and elsewhere [36,37]. Motivation can be defined as an individual's degree of willingness to exert and maintain an effort towards organisational goals [38]. Misalignment of goals was evident in our study, for example in attitudes towards in-service training: opportunities were found to negatively affect motivation of staff not selected and this was as much due to the loss of opportunity for extra payments as for learning. This complements Aitken's [36] finding in Nepal that training was seen as a way of providing allowances without having the desired effect of improving performance. Supervision needs to be sensitive to organisational and worker goals, and this involves establishing a relationship of trust between management and workers [39]. Manzi *et al *[40] found health worker experiences of supervision at health facilities in Tanzania varied and depended upon the nature of supervision as well as the manner of the supervisor. These findings emphasize the need for human resource management training as a priority in healthcare systems [35].

The perception of pressure from patients has been found to influence prescribing behaviour for diarrhoeal disease in other developing country settings [23,24], and in Pakistan clinicians reported prescribing antidiarrhoeals because parents demanded drugs and antidiarrhoeals could be used as a placebo [41]. Interestingly, patient expectations have been found to differ from how they are perceived by clinicians in the case of antimalarial prescribing in Tanzania where patients were not observed to demand antimalarials directly, not to be more satisfied if they had received an antimalarial prescription when interviewed on exit from consultations [42], echoing findings relating to antibiotic prescribing in Europe and the US [43-45]. Malaria has been described as a socially acceptable disease, which may influence the decision to diagnose malaria in preference to alternative diseases [46]. Group level clinician interventions have been successful in changing perceptions of patient preferences. For example, Hadiyono *et al *[47] included discussions of prescribers' assumptions about patient beliefs and how this affects their behaviour in interactional group discussions which were effective in reducing injection prescriptions in Indonesia. Public health messages that promote the importance of alternative causes of illness may also affect both perceived and actual patient pressure.

This study's finding that peer pressure affected practice echoes findings amongst general practitioners in England who reported discomfort in prescribing related to the fear of looking less competent to peers or of breeching an agreed or understood management policy [48]. The influence of peers has been found to have an impact on behaviour change [49] been used to change clinical behaviour. Bandura [50] argues that behaviour change is heavily contingent upon self-efficacy, the perception of one's ability to perform a behaviour, and that peer group discussion has the potential for increasing a doctor's self-efficacy. This has been borne out in the success of interactive workshops or discussion sessions to change prescribing behaviour, particularly for antibiotics, in a variety of settings [51-53]. That these interventions take place in peer groups allows group discourses to change, creating a new anchor for individual behaviour.

Guidelines of malaria diagnosis and treatment are known to be confusing [54] and this was reflected in this study. Students were found to be taught guidelines alongside adopting collective mindlines, resulting in conflicting messages. Suggestions for guidelines that encompass malaria as well as alternative diseases and co-morbidity have recently been outlined [55] and the adoption of clearer guidelines will be an essential precursor for improving practice [56]. Guidelines need to emphasize differential diagnoses, and potentially a symptomatic rather than disease driven approach. Reliance on guideline dissemination and in-service basic training alone, however, has not been greatly successful in improving malaria treatment decisions [13,57], nor in changing other clinical behaviours [58,59]. Motivation to disseminate, learn, and follow these guidelines will still need to be addressed.

The study was conducted at hospitals, where malaria testing is routinely available. By contrast, in health centre and dispensary settings laboratory facilities are less often available, over-diagnosis of common diseases such as malaria is inevitable. The potential introduction of rapid diagnostic tests to these settings may change this and in-depth studies are needed to understand the diagnostic and prescribing behaviour of health workers in these settings where the majority of the population first encounter the public health system.

Recognition and treatment of malaria is of major importance in Africa where it is a key cause of morbidity and mortality. Its importance is well established and the development era has seen an increase in the emphasis on malaria, particularly in relation to poverty. This is exemplified by its prominence in the UN Millennium Development Goals. It has been argued that the identification of malaria as a key underlying 'problem' is due to the availability of a technical 'solution' [60]. As donor-recipients, Tanzanian health officials are under pressure to channel efforts into malaria-specific activities. This has a knock-on effect throughout the healthcare system of emphasizing malaria to the detriment of other diseases, as has been demonstrated in this paper. Whilst clinicians are right to fear the consequences of missing malarial disease, alternative causes of symptoms comparable to those indicating malaria are likely to be bacterial [5], and may be secondary to HIV [61] but these diagnoses were infrequently made. The message that the consequences of missing alternative diagnoses can be as severe as missing malaria is currently overshadowed, but needs to be conveyed both in clinician-centred interventions and in wider arenas affecting decision makers, through both public and medical media.

While several studies have pointed to the individual influence of each of these factors on prescribing behaviour, this study suggests that clinical decision-making for febrile illness is affected by these spheres of influence through rationales or 'mindlines' that are distinct from clinical guidelines. The mindlines identified were that malaria is an easier diagnosis than alternative diseases; that malaria is a more acceptable diagnosis; and that to miss a malaria diagnosis is indefensible. To improve clinical decision-making in this context, interventions need to target the factors that influence these mindlines. The social nature of these spheres of influence requires group as well as individual level interventions.

## Conclusion

The evidence of the over-diagnosis of malaria in febrile disease in Africa is now overwhelming. Simple solutions such as providing better diagnostics do not in themselves lead to a reduction in over-diagnosis; since it is now clear that malaria over-diagnosis occurs, an understanding of *why *it occurs is now needed. The findings of this study indicate that whilst patients stand to benefit clinically from being considered as non-malarial in the face of negative test results for malaria and even without malaria testing, rationales for clinicians to diagnose malaria and prescribe antimalarials appear to outweigh this. These shared rationales, or 'mindlines', are drawn from different spheres of influence including diagnostic support, perceived patient preferences, peer pressure and disease promotion and training. Clinical decisions are, therefore, entrenched in mindlines that are collectively reinforced and that have predominantly been constructed by social influences, in conjunction with, or instead of, guidelines. Interventions to change clinical decision-making need to focus on the influencing factors identified and need to use methods that address the collective nature of mindlines currently in use.

## Authors' contributions

CC carried out the ethnography, analysis, and drafted the paper. CJ contributed to the study design and was involved in analysis and drafting of the paper. GB and KJ assisted with data collection and critically revised the paper. HR contributed to the study design and critically revised the paper. CW contributed to the study design and analysis and was involved in drafting and revising the paper.
